# Prevalence of human cystic echinococcosis in the towns of Ñorquinco and Ramos Mexia in Rio Negro Province, Argentina, and direct risk factors for infection

**DOI:** 10.1186/s13071-021-04753-y

**Published:** 2021-05-19

**Authors:** Leonardo Uchiumi, Guillermo Mujica, Daniel Araya, Juan Carlos Salvitti, Mariano Sobrino, Sergio Moguillansky, Alejandro Solari, Patricia Blanco, Fabiana Barrera, Janete Lamunier, Marcos Arezo, Marcos Seleiman, Zaida E. Yadon, Francesca Tamarozzi, Adriano Casulli, Edmundo Larrieu

**Affiliations:** 1 Hospital “Artémides Zatti”, Viedma, Provincia de Río Negro Argentina; 2 Coordinación de Salud Ambiental, Ministerio de Salud, Viedma, Provincia de Rio Negro Argentina; 3 Hospital “Ramón Carrillo”, San Carlos de Bariloche, Provincia de Río Negro Argentina; 4 Facultad de Medicina, Universidad del Comahue, Cipolletti, Provincia de Río Negro Argentina; 5 Hospital “Raul Fernicola”, Valcheta, Provincia de Rio Negro Argentina; 6 Hospital Area Programa de Ramos Mexia, Ministro Ramos Mexía, Argentina; 7 Hospital Area Programa de Ñorquinco, Ñorquinco, Argentina; 8 Instituto de Investigaciones Epidemiológicas, Academia Nacional de Medicina, Buenos Aires, Argentina; 9 WHO Collaborating Centre for the Epidemiology, Detection and Control of Cystic and Alveolar Echinococcosis, Department of Infectious Diseases, Istituto Superiore Di Sanità, Rome, Italy; 10 European Union Reference Laboratory for Parasites, Department of Infectious Diseases, Istituto Superiore Di Sanità, Rome, Italy; 11 Facultad de Ciencias Veterinarias, Universidad Nacional de La Pampa, General Pico, Provincia de La Pampa Argentina; 12 Escuela de Veterinaria, Universidad Nacional de Rio Negro, Choele Choel, Provincia de Rio Negro Argentina

**Keywords:** Cystic echinococcosis, *Echinococcus granulosus* (*s.l.*), Ultrasound screening, Epidemiology, Risk factors

## Abstract

**Background:**

Cystic echinococcosis (CE) is a parasitic zoonosis caused by infection with the larval stage of *Echinococcus granulosus* (*s.l.*). This study investigated the prevalence and potential risk factors associated with human CE in the towns and rural areas of Ñorquinco and Ramos Mexia, Rio Negro province, Argentina.

**Methods:**

To detect abdominal CE cysts, we screened 892 volunteers by ultrasound and investigated potential risk factors for CE using a standardized questionnaire. Prevalence ratio (PR) with 95% confidence intervals (CI) was used to measure the association between CE and the factors investigated, applying bivariate and multivariate analyses.

**Results:**

Abdominal CE was detected in 42/892 screened volunteers (4.7%, 95% CI 3.2–6.1), only two of whom were under 15 years of age. Thirteen (30.9%) CE cases had 25 cysts in active stages (CE1, CE2, CE3a, according to the WHO Informal Working Group on Echinococcosis [WHO-IWGE] classification). The most relevant risk factors identified in the bivariate analysis included: living in rural areas (*P* = 0.003), age > 40 years (*P* = 0.000), always drinking water from natural sources (*P* = 0.007), residing in rural areas during the first 5 years of life (*P* = 0.000) and having lived more than 20 years at the current address (*P* = 0.013). In the multivariate final model, the statistically significant risk factors were: frequently touching dogs (*P* = 0.012), residing in rural areas during the first 5 years of life (*P* = 0.004), smoking (*P* = 0.000), age > 60 years (*P* = 0.002) and living in rural areas (*P* = 0.017).

**Conclusions:**

Our results point toward infection with CE being acquired since childhood and with constant exposure throughout life, especially in rural areas with a general environmental contamination.

**Graphic Abstract:**

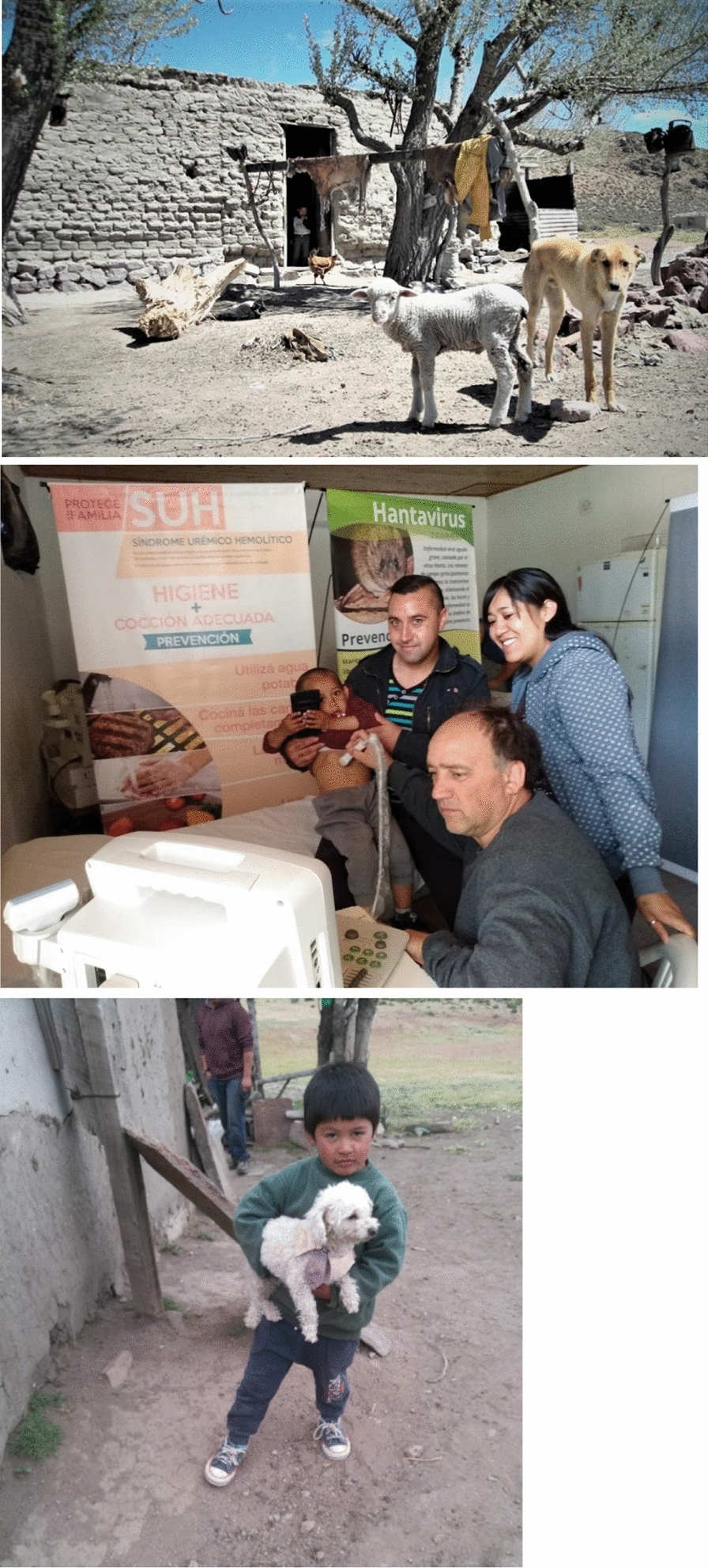

**Supplementary Information:**

The online version contains supplementary material available at 10.1186/s13071-021-04753-y.

## Background

Cystic echinococcosis (CE) is a parasitic zoonosis, mainly prevalent in rural areas, caused by infection with the larval stage (metacestode) of *Echinococcus granulosus* (*s.l.*). The transmission cycle of the parasite requires two mammalian hosts, a definitive canid host (usually a dog) for the development of the adult tapeworm and an intermediate host (usually livestock, mainly sheep), where the parasitic larval stage develops as a cyst in internal organs [1, 2]. The parasite transmission cycle is fostered by human practices of feeding dogs with raw offal containing infective *E. granulosus* cysts after home slaughtering [3].

Humans behave as accidental dead-end intermediate hosts for the cestode, getting infected through ingestion of parasite eggs, without participating in its biological cycle [4]. Pathways of transmission such as food, contaminated water, direct contact or playing with dogs are classically mentioned as sources of human infection and biologically plausible risk factors [4]. However, there are no rigorously gathered data on the contaminated matrices and pathways of transmission of infection to humans and their relative contribution in different transmission areas.

In South America, CE is a public health problem, particularly in Argentina, Brazil, Chile, Uruguay and Peru [2, 5]. In Argentina, in the Rio Negro province, CE burdens the health system with high costs for patient care [6]. In this province, the CE Control Programme, launched in 1980 by the Ministry of Health, is based on Primary Health Care and One Health strategies that include deworming of dogs with praziquantel, sheep vaccination, health education, early diagnosis in humans by means of regular ultrasound (US) screening campaigns and medical and/or surgical treatment of infected individuals [5, 7, 8]. Since 1997, the CE Control Programme has used abdominal US screening of the asymptomatic school children population [9] and, although not routinely, also of adults [9, 10].

In 2019, the CE Control Programme, in partnership with the Universidad Nacional de Río Negro and the Italian Istituto Superiore di Sanità (project coordinator), participated in the collaborative, multicenter study “Molecular-Epidemiological Studies on Pathways of Transmission and Long Lasting Capacity Building to Prevent Cystic Echinococcosis” (PERITAS), funded by the European Union through the EU-LAC Health project. Other project’s partners included the Consejo Superior de Investigaciones Científicas (Spain), the Instituto de Salud Carlos III (Spain), the Universidad Austral de Chile (Chile) and the Universidad Peruana Cayetano Heredia (Peru). PERITAS aims to (i) conduct abdominal US surveys to assess the prevalence of abdominal CE and identify clusters of infection in the all-age population of selected areas in Argentina, Chile and Peru; (ii) carry out environmental sampling for the detection of *E. granulosus* eggs; (iii) identify the potential risk factors associated with the transmission of *E. granuosus* to humans.

Here, we present the results of the study carried out to estimate the prevalence of abdominal CE and to identify specific risk factors associated with infection transmission of *E. granuosus* to humans in the towns of Norquinco and Ramos Mexia, Rio Negro province, Argentina.

## Methods

### Study design

We conducted a cross-sectional, community-based study on volunteer participants by means of abdominal US. Participants were also interviewed using a structured standardized questionnaire focused on potential risk factors and habits, including frequencies of their acting, which may favor human infection (Additional file 1: Annex S1).

### Work area

**Fig. 1:**
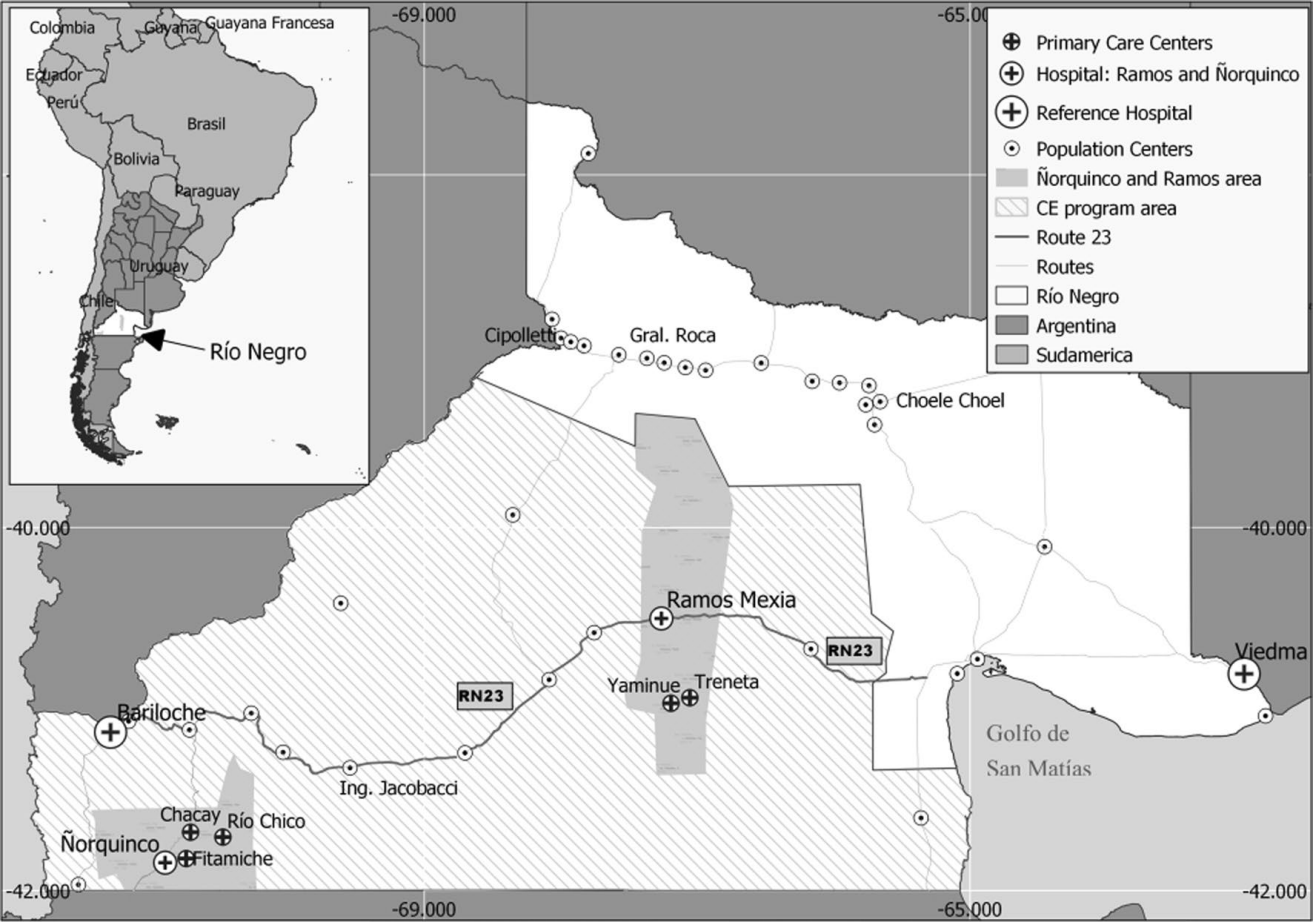
Geographical distribution of reference hospitals, rural hospitals, primary health care centers (PHCC) and rural areas without PHCC along the National Route 23 in Rio Negro, Argentina

The study areas were the towns of Ñorquinco and Ramos Mexia as well as their surrounding rural areas in the Rio Negro province (Fig. 1), with a population of nearly 3200 people (1800 in Ñorquinco and 1400 in Ramos Mexia), as estimated by the primary health care services of the local hospitals, based on records from home visits by rural health workers. The majority of the population lives in urban areas. The rural population resides in small clusters (27 inhabitants in Treneta) or in dispersed settlements around Ñorquinco (511 people).

Both towns have a small rural hospital staffed by two general practitioners, who coordinate the rural Primary Health Care Centers (PHCC) and the medical care posts (Fig. 1). This health care network is the only health care provider in each area.

Ñorquinco area is located in the western mountain region of the province, extending over approximately 5706 km^2^, with ideal conditions of humidity, temperature and vegetation for the survival of *E. granulosus* eggs. Ramos Mexia area extends over approximately 9680 km^2^ and is located in the east of the province, in the Patagonian steppe, where the very dry and hot summer could limit the survival of *E. granulosus* eggs. Both study areas have the conditions for the maintenance of the parasite life cycle: a high proportion of the population having a low socioeconomic status, high number of families owning several dogs, the predominance of sheep farming for wool production and the practice of home slaughtering of sheep and goats and feeding dogs with raw viscera [8, 11].

Both areas are targeted by the CE Control Programme, since 1980 in Ñorquinco and 1986 in Ramos Mexia. Rural health workers are responsible for health education during house-to-house visits and for the distribution of praziquantel for dogs deworming four times a year. Deworming is usually demanded of the dog’s owner. Veterinary teams are responsible for the surveillance of infection in dogs (originally with arecoline test and currently with coproELISA) and in sheep (by necropsy). Surveillance of CE in the human population includes the regular performance by general practitioners of US screenings in schoolchildren and the systematic registration of identified cases [7, 8, 12]. As a result of the Control Programme activities, during the period 1980–1996, 1720 new CE cases were identified in the province (average 101 cases per year), while in 2006–2016 the identified CE cases dropped to 478 (average 43 cases per year) [13].

Ñorquinco always presented higher prevalence rates than Ramos Mexia. For example, in 2003 the percentage of sheep farms with infected dogs (tested with coproELISA test and/or PCR) was 11.8% in Ñorquinco and 0% in Ramos Mexia, while the prevalence rate in schoolchildren (assessed by US) was 1.0% in Ñorquinco and 0.3% in Ramos Mexia [14]. In both areas, prevalence shows a decreasing trend [8, 12].

### Population screening

Health workers from each rural hospital made house-to-house visits to explain the aim of the study and invite the people to participate. The US screening was conducted on the convenience sample of people of all ages and both sexes who volunteered to participate. Each adult participant, or a parent or legal representative in case of minors, signed the informed consent form and filled the questionnaire (Additional file 1: Annex S1) before being examined by US. One or two US machines were installed in the hospitals while other machines were rotated among the rural PHCC and schools. In total, five machines were available in Ramos Mexia and six in Ñorquinco.

The Focused Assessment with Sonography for Echinococcosis (FASE) protocol [13] was used for abdominal examinations. CE diagnosis and cyst staging were carried out according to the WHO Informal Working Group on Echinococcosis (WHO-IWGE) Expert Consensus document [14]. CE case definition and clinical management were applied according to the Provincial Norms of Diagnosis and Treatment of Cystic Echinococcosis, approved by the Resolution 2624–2018 of the Ministry of Health, Rio Negro Province [13, 15]. Briefly, in this document, a CE case is defined in the presence of pathognomonic features of CE on imaging, or macro- or microscopic identification of any component of the CE cyst in a specimen, or morphological changes of the cyst on imaging or seroconversion after specific medical treatment. A suspect CE case is defined in the presence of only one serological positive test (different than Western blot) or the presence of a cyst without pathognomonic imaging features. In case of uncertain diagnosis, the participants were referred to the hospitals for further advanced imaging testing by US, computed tomography (CT), or magnetic resonance imaging (MRI), as needed. Results of hospital-based examinations were retrieved, and to finally classify them as confirmed or not, the epidemiological information of these subjects was processed according to the final diagnosis. All CE identified and confirmed cases were entered in the CE Control Programme database, and medical records were checked to verify whether the case was new or had a previous diagnosis of CE.

### Data collection

Epidemiological data were obtained by interviewing the study participants using a standardized questionnaire (Additional file 1: Annex S1). The information collected included demographic data (age, sex, place of residence, time since residing the place, place of living during the first 5 years of life), having any relative with CE in the household and personal behaviors possibly associated with ingestion of *E. granulosus* eggs (owning and touching dogs, growing and eating raw and unwashed vegetables, source of drinking water, habits related to hand washing, smoking, use of toothpick, or habit of chewing).

### Analysis of data

The epidemiological, medical records and screening data were transferred to the database created in Microsoft Excel® 2.0 (Redmond, WA, USA) for all subsequent processing activities and quality control.

CE prevalence with 95% confidence interval (CI) was calculated according to age, sex, place of residence and previously known CE infection status. Place of residence during the first 5 years of life was classified into an urban or rural area, and the years of living at the current address were categorized in < 5 years, 5–10 years, 11–20 years and > 20 years.

The association between CE infection and the studied variables was estimated using prevalence rate (PR) with a 95% CI through bivariate and multivariate analyses using STATA™ 12.0. The variable “eating raw unwashed vegetables” was not included in the analysis because all cases stated that they always cooked or washed vegetables before consumption. A multivariate analysis binomial regression model was applied, starting with inclusion of all factors that had a *P*-value ≤ 0.25 in the bivariate analysis and other risk factors selected *a priori*, based on published data (dog ownership in the past 5 years and touching dogs). A manual stepwise backward selection was used to define the final model. We used the lowest Bayesian information criterion (BIC) to identify the best-fitting model given the data collected. Only variables yielding a two-tailed *P*-value < 0.05 were considered significant and included in the final model.

## Results

### Population screening

A total of 892 volunteers participated in the survey, representing 28% of the 3200 total population living in the study areas. Of them, 42/892 (4.7%, 95% CI 3.2–6.1) had abdominal CE, among whom 13 CE cases (30.9%) had 25 cysts in active stages (CE1, CE2, CE3a).

Based on the place of residence, 309/1800 (17.2%) inhabitants were examined in Ñorquinco with 13 CE cases detected (4.2%, 95% CI 1.8–6.6) in volunteers aged 10–83 years. Of these, six were living in urban areas (6/231; 2.6%, 95% CI 0.95–5.5) and seven in rural areas (7/78; 9.0%, 95% CI 3.6–17.6). In Ramos Mexia, 583/1,400 (41.6%) inhabitants were examined, with 29 CE cases (4.9%, 95% CI 3.1–6.8) detected in participants aged 2 to 81 years old. Of these, 18 were living in urban areas (18/451; 4.0%, 95% CI 2.4–6.3) and 11 in rural areas (11/132; 8.3%, 95% CI 4.2–14.4) (Table 1).

**Table 1:** Demographic and risk factors for abdominal cystic echinococcosis in Ñorquinco and Ramos Mexia, Rio Negro, Argentina, 2019

Variables	POP**	US+* (*n* = 42)		US−* (*n* = 850)		PR (95%CI)	*P*-value
	*N*	*N*	%	N	%		
Residence							
Norquinco	309	13	30.9	296	34.8	1	
Ramos Mexia	583	29	69.1	554	65.2	1.18 (0.62–2.24)	0.608
Total	892	42		850			
Living location							
Urban	682	24	57.1	658	77.4	1	
Rural	210	18	42.9	192	22.6	2.43 (1.34–4.40)	0.003
Total	892	42		850			
Age groups (years)							
0–19	317	3	7.1	314	37.0	1	
20–39	219	6	14.3	213	25.1	2.89 (0.73–11.46)	0.130
40–59	244	14	33.3	230	27.2	6.0 (1.7–20.8)	0.004
> 60	110	19	45.3	91	10.7	18.2 (5.5–60.5)	0.000
Total	890	42		848			
Sex							
Female	514	18	42.9	496	58.4	1	
Male	378	24	57.1	354	41.6	1.81 (0.99.29)	0.051
Total	892	42		850			
Dog ownership in the past 5 years							
No	70	2	5.1	68	9.4	1	
Yes	692	37	94.9	655	90.6	1.87 (0.46–7.60)	0.381
Total	762	39		723			
Touch dogs							
Never	175	6	15.0	169	20.8	1	
Rarely	146	9	22.5	137	16.7	1.79 (0.65–4.93)	0.255
Frequently	535	25	62.5	510	62.5	1.36 (0.56–3.26)	0.488
Total	856	40		816			
Eat vegetables grown in a kitchen garden							
Never	510	23	56.1	487	59.5	1	
Rarely	213	11	26.9	202	24.7	1.14 (0.56–2.30)	0.705
Frequently	136	7	17.0	129	15.8	1.14 (0.50–2.60)	0.754
Total	859	41		818			
Nail biting							
No	654	36	85.7	618	75.0	1	
Yes	212	6	14.3	206	25.0	0.51 (0.21–1.20)	0.125
Total	866	42		824			
Smoking							
No	691	33	78.6	658	88.4	1	
Yes	95	9	21.4	86	11.6	1.98 (0.97- 4.01)	0.057
Total	786	42		744			
Use toothpicks or chew blade of grass							
No	581	26	63.4	555	73.5	1	
Yes	215	15	36.6	200	26.5	1.55 (0.84- 2.88)	0.158
Total	796	41		755			
Hand washing before cooking							
Never	795	39	92.9	756	94.5	1	
Every time	47	3	7.1	38	4.7	1.30 (0.41–4.05)	0.650
Type of drinking water							
Always public network or bottled	636	22	52.4	614	73.3	1	
Occasionally non-potable	108	8	19.0	100	11.9	2.14 (0.97–4.68)	0.057
Always non-potable	136	12	28.6	124	14.8	2.55 (1.29–5.02)	0.007
Total	880	42		838			
Have relatives with CE in the household							
No	559	29	76.3	530	75.8	1	
Yes	179	9	23.7	170	24.2	0.96 (0.46–2.00)	0.933
Total	738	38		700			
Resided in rural areas during the first 5 years of life							
No	418	8	20.5	410	58.5	1	
Yes	322	31	79.5	291	41.5	5.03 (2.34–10.79)	0.000
Total	740	39		701			
Years lived in the area of the current address							
< 5 years	133	1	2.5	132	17.0	1	
5 to 10 years	139	5	12.5	134	17.0	4.78 (0.56–40.46)	0.151
11 to 20 years	209	3	7.5	206	26.0	1.90 (0.20–18.18)	0.574
> 20 years	346	32	77.5	315	40.0	12.26 (1.69–88.96)	0.013
Total	827	41		787			

*PR* prevalence rate

^*^U+: CE cysts on abdominal ultrasound; US−: no CE cysts on abdominal ultrasound; POP: number of people studied

**Fig. 2:**
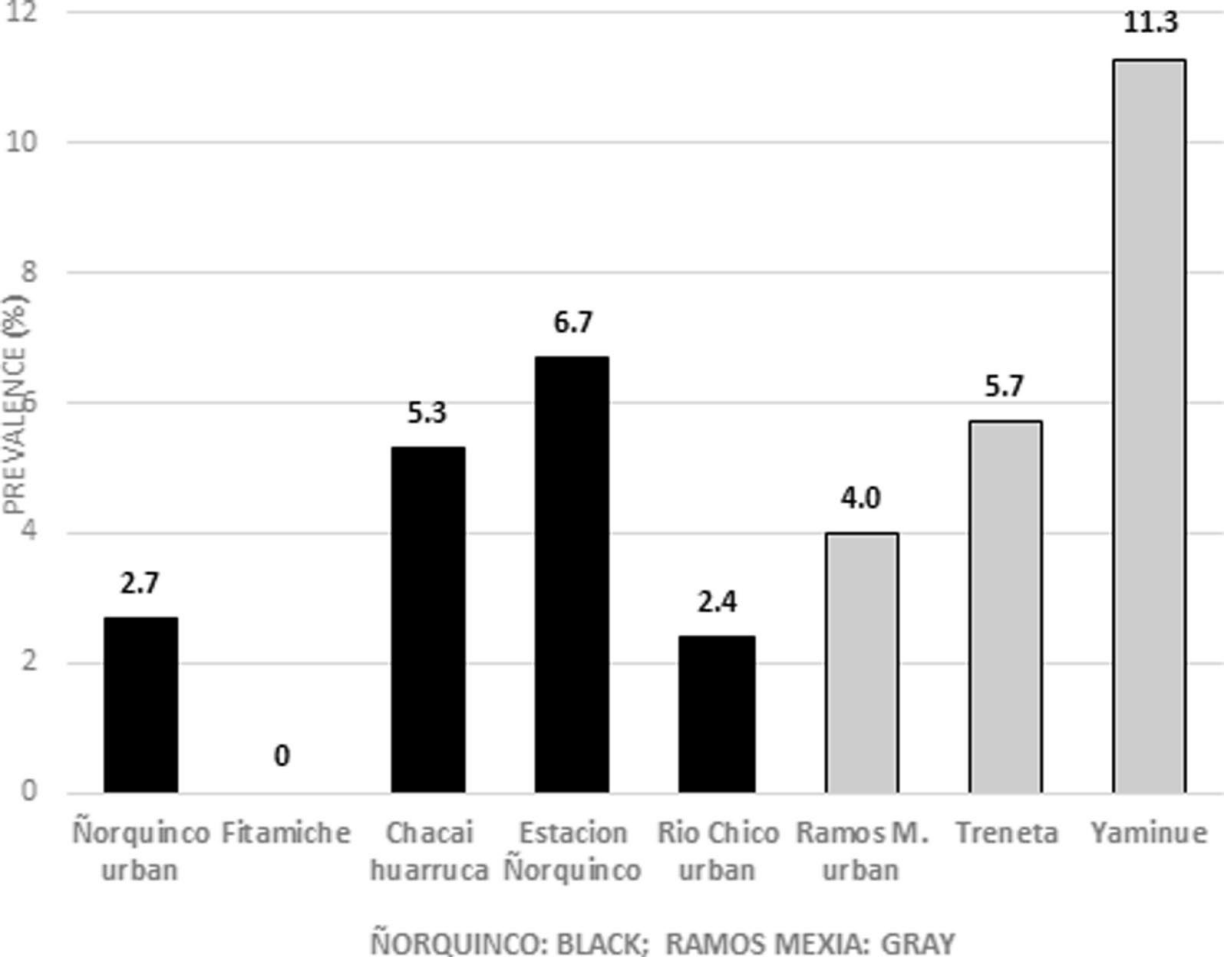
Prevalence of abdominal CE according to place of residence in Ñorquinco and Ramos Mexia areas, Rio Negro, Argentina, 2019

The prevalence of abdominal CE was similar between Ñorquinco and Ramos Mexia (*P* = 0.60); however, the prevalence rate was significantly higher in rural areas than in urban areas (*P* = 0.003) (Table 1). Figure 2 shows the prevalence of CE by place of residence.

The majority of the study participants were women (58% women, 42% men). The proportion of CE cases was higher in men than in women, the difference being statistically significant (*P* = 0.047) (Table 1).

The mean age of the study population was 34 ± 21 years. The age was significantly different (*P* < 0.0001) between volunteers with abdominal CE (mean 57 ± 19 years) and without CE (mean 33 ± 20 years). The analysis of CE prevalence by age group (categorized in 0–19, 20–39, 40–59, > 60 years) showed a statistically significant increase with age.

All individuals with CE cysts in CE1 stage (uniloculated, fluid-filled cyst considered the first developmental stage of the metacestode in the intermediate host), independently of length of living in the study area, had been living in rural areas during their first 5 years of life, and 79.5% of CE cases, regardless of the cyst stage, also lived the first 5 years of their life in rural areas.

Of the 42 abdominal CE cases, 31 were diagnosed during the project screening and classified as new cases (31/892; 3.5%, CI 2.2–4.7), while 11 (11/892; 1.2%, 95% CI 0.4–2.0) had been previously diagnosed in a clinical setting or during the activities of the CE Control Programme. Of the detected cases, 83.3% (35/42) were born before the implementation of the CE Control Programme.

The number of detected CE cysts was 58 (mean 1.4 cysts per case): 52 (89.7%) in the liver, 5 (8.6%) in the spleen and 1 (1.7%) in the kidney.

**Fig. 3:**
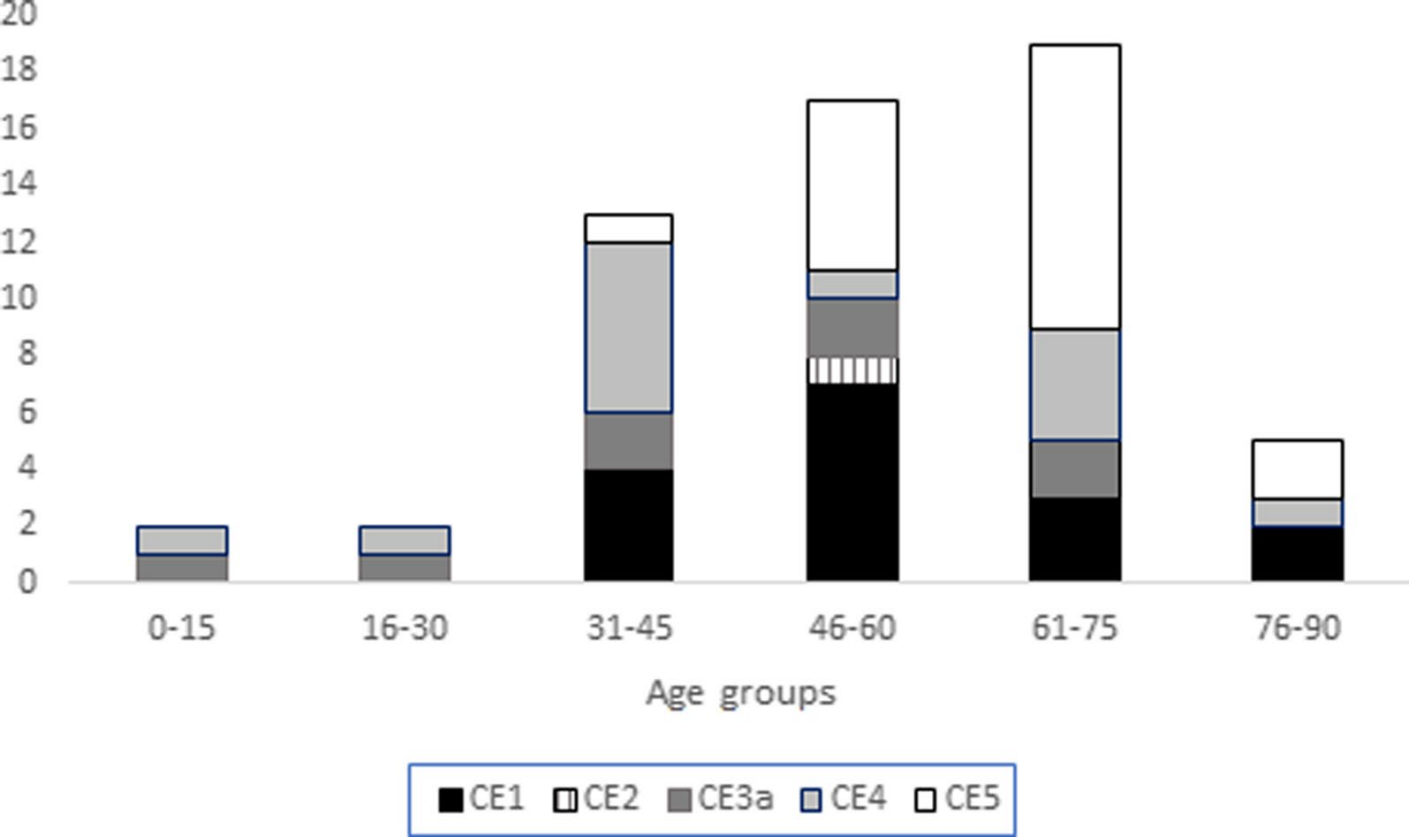
Distribution of the CE cyst stages (58 cyst detected in 42 cases) according to the WHO Informal Working Group on Echinococcosis classification by age groups. Ñorquinco and Ramos Mexia, Rio Negro, Argentina, 2019. CE1, unilocular fluid-filled; CE2, with daughter cysts; CE3a, with detached parasitic layers; CE4, with solid content; CE5, with solid content and calcified walls. CE1–CE2 stages are considered active cysts, CE3a transitional (50% being biologically active and 50% inactive), while CE4–CE5 cysts are considered inactive

According to the WHO-IWGE classification, 16 (27.6%) cysts were in the CE1 stage, 1 (1.7%) CE2, 8 (13.8%) CE3a, 14 (24.1%) CE4 and 19 (32.8%) CE5. Subjects with cysts in the CE1 stage were 32–80 years of age. Notably, 31.5% of cysts in the CE1 stage, and 51.5% of cysts in the CE4 and the CE5 stages were in the > 60-year age group (Fig. 3). The prevalence of CE among children was 0.9% (95% CI 0.1–3.0); of the 234 screened children aged less than 15 years old, two had abdominal CE (4.8% of all cases), one newly identified with a stage CE3a cyst and one already known with a CE4 cyst.

### Risk factors analysis

As shown in Table 1, the demographic characteristics and risk factors significantly associated with CE in the bivariate analysis were being ≥ 40 years old, having lived in a rural areas during the first 5 years of life, living in a rural area, always drinking non-potable water, frequently touching dogs and smoking.

The analysis of the relationship of the place of birth and the time of residence at the same address may give further insight into the relationship of these variables. For instance, a 63-year-old patient had lived < 5 years in her current address (Treneta, a rural area) but came from another endemic rural area (Yaminue). Five cases with < 10 years spent in their current domicile had lived their first 5 years of life in endemic rural areas. Only one 10-year-old patient with CE had lived all his life in an urban area (Rio Chico) but visited the family farm in a nearby rural area frequently.

Table 2 shows the variables that remained in the final model.

**Table 2:** Binomial logistic regression model of risk factors for abdominal CE in Ñorquinco and Ramos Mexia, Rio Negro 2019

Variables	PR (95%CI)	*P*-value
Age group (years)		
0–19	1	
20–39	0.37 (0.31–5.33	0.713
40–59	1.27 (0.65–7.87)	0.197
> 60	3.12 (2.01–23.4)	0.002
Place of residence		
Urban	1	
Rural	2.28 (1.1–2.8)	0.017
Touch dog		
Never	1	
Rarely	1.76 (0.88–9.99)	0.078
Frequently	2.50 (1.30–9.29)	0.012
Resided in rural areas during the first 5 years of life		
No	1	
Yes	2.89 (1.47–3.87)	0.004
Smoking		
No	1	
Yes	4.36 (1.67–3.99)	0.000

*n* = 692

*PR* prevalence ratio

## Discussion

The US abdominal screening revealed a 4.7% overall prevalence of asymptomatic human abdominal CE in the Departments of Ñorquinco and Ramos Mexia, Rio Negro province, with a 1.5% prevalence of CE in active stages. Thus, despite control efforts made in the province since 1980, the overall prevalence of CE is still relatively high, indicating that CE remains as a public health problem in the area. Nevertheless, surveillance data in Rio Negro show a sustained decrease of human CE cases associated with the implementation of control measures [5, 8]. In accordance, in our survey the number of CE cases in children under 15 years of age are extremely low.

In the general population, the prevalence of CE was significantly lower in the Departments of Ñorquinco and Ramos Mexia compared to results of studies conducted in other areas of Rio Negro in areas with similar geographical and epidemiological characteristics: overall prevalence of 8.3% in Pilcaniyeu and Comallo in 1984 [10] (*P* = 0.00001) and prevalence of 7.1% in Ingeniero Jacobacci in 2009 [9].

The higher prevalence rate of abdominal CE in subjects born before the implementation of the control measures, particularly those who resided during their first 5 year of life in rural areas, suggests that the transmission may have occurred during childhood or youth, consistent with previous findings [11, 16]. On the other hand, the finding of a high percentage of CE1 cyst stages (27.6%) in adults may arguably reflect a recent transmission in adults. Nevertheless, it may also be the result of the stability in the CE1 stage of an infection acquired in the past. This is consistent with previous results reported from Kenya and Morocco, with 87.3% of cysts remaining in the same stage for years [15] and from Rio Negro itself, where 18.8% of CE1 and CE3 cyst stages in children remained unaltered even after 10 years of follow-up [17].

Ñorquinco and Ramos Mexia presented similar prevalence rates, but this was significantly higher in rural (8.57%) than urban areas (3.51%; *P* = 0.003). This result is consistent with CE being a rural zoonosis and overall with the results of other studies carried out in the area [9, 10]. Our data show a significant risk of CE related to geo-demographic factors (increasing age, having lived in rural areas during the first 5 years of life, currently living in rural areas), strict contact with the definitive host (frequently touching dogs) and habits linked to hand-to-mouth transmission (smoking).

The increased risk of CE associated with frequently touching dogs is consistent with the fact that in the study area dogs have access to parasitized viscera and humans cohabit and interact with dogs from birth. Smoking, a novel finding of our study compared to what was explored in previous studies, was found to be associated with CE and plausibly transmitted through fostering a “hand-to-mouth” transmission mechanism. All smokers were ≥ 17 years old. Except for frequently touching dogs and smoking, no other variables was related to the specific behavior of subjects putting their hands in the mouths were associated with CE.

Drinking non-potable water was associated with CE only in the bivariate analysis. Although not significantly associated in the final model, inhabitants of these rural areas frequently and early in life drink non-potable water that may be contaminated. Thus, their contact with *E. granulosus* eggs occurs early in life and is likely intense especially in childhood. This higher exposure in childhood is supported by results from some studies [16, 18, 19] but not by others [20, 21]. It is possible that different cultural habits may explain these differences.

The use of non-potable water as a risk factor for CE is controversial. In South America, it has only been described as a significant risk factor for CE in Peru, Uruguay [22, 23] and Rio Negro, Argentina, in bivariate analyses [24], while in Europe no such association was found [4]. A recent systematic review and meta-analysis supported the hypothesis that having contact with dogs and consumption of non-potable water (and to a lesser extent of contaminated food) could be pathways of CE transmission [25]. However, at present there is not enough experimental evidence of the contamination of these matrices by parasite eggs, which may support their role as a source of transmission [4].

One of the strengths of this study is the large number of subjects screened (28% of the population); this was allowed by using the routine district health services for their implementation and the considerable number of US machines available, which facilitated the access of the population to the screening activities. Furthermore, differently, from what is generally done in studies of this type, our infection risk factor investigation included variables evaluating behaviors and their frequency, potentially inducing the ingestion of *E. granulosus* eggs rather than questions related merely to the perpetuation of the parasite cycle. However, as recently highlighted [26], risk factor evaluation for chronic diseases such as CE, which have long latency and no acute symptoms that can induce the patient to consultation, has limitations particularly when using a cross-sectional design.

The study had several limitations and potentials for bias. First, it was carried out in an area where a CE Control Programme has been implemented for decades and in the context of which health education campaigns were carried out regularly in schools and through the media. This may have not only influenced a change in people’s behaviors but also induced them to answer with what they thought was expected by the researcher. Selection bias also may have arisen because the screened sample was self-selected because of the voluntary nature of their participation. The difficulties of mobilizing the community from remote rural areas to the US screening post, although mitigated by the displacement of screening sites, may have also contributed to selection bias influencing the result in either direction. Finally, training of the interviewers and pre-testing of the questionnaire, which were originally envisaged, could not be carried out in time to be compatible with the scheduled fieldwork. This may have introduced some problems with the collection of the information, leading to some missing data for some variables (from 1–17%), thus preventing the availability of a full dataset for inclusion in the multivariate analysis.

## Conclusions

The result of this study shows that the prevalence of CE in Rio Negro is decreasing, likely because of the measures included in the CE Control Programme. However, CE continues to be a relevant zoonosis in the area. Although transmission to humans has been considerably reduced through canine deworming and health education strategies associated with the systematic search for asymptomatic carriers, especially in the school children population, cases continue to be detected in adults (some with cysts in active stages), and it remains to be determined whether these are recent or old infections.

Feasible primary prevention measures aimed at avoiding the ingestion of *E. granulosus* eggs can help to reduce the burden of CE. The identification of the main sources of infection and habits which may increase the risk of exposure may allow interventions that can be tailored to each specific population, including those relevant for health education messages [4, 27]. Factors associated with a higher risk of CE found here suggest that people acquire the infection in rural areas, a proxy of environmental contamination, over time while residing in such an environment, rather than as the result of very specific habit(s); also, it is likely that infection is linked with direct or indirect contamination of the hands more than ingesting eggs through contaminated food and water, as suggested also in other works [28]. This is relevant to control strategies, as it is important to identify risks that can be reduced by feasible and practical interventions.

## Supplementary Information

**Additional file 1: Annex S1.** Questionnaire.

## Data Availability

The data used to support the results of this study are available from the corresponding author upon request.

## References

[1] Otero-Abad B, Torgerson PR (2013). A systematic review of the epidemiology of echinococcosis in domestic and wild animals. PLoS Negl Trop Dis.

[2] Craig PS, Hegglin D, Lightowlers MW, Torgerson PR, Wang Q (2017). Echinococcosis: control and prevention. I. Echinococcus and Echinococcosis. Adv Parasitol.

[3] Larrieu E, Gavidia CM, Lightowlers MW (2019). Control of cystic echinococcosis: background and prospects. Zoonoses Public Health.

[4] Tamarozzi F, Akhan O, Cretu CM, Vutova K, Fabiani M, Orsten S (2019). Epidemiological factors associated with human cystic echinococcosis: a semi-structured questionnaire from a large population-based ultrasound cross-sectional study in eastern Europe and Turkey. Parasit Vectors.

[5] Pavletic CF, Larrieu E, Guarnera EA, Casas N, Irabedra P, Ferreira C (2017). Cystic echinococcosis in South America: a call for action. Rev Panam Salud Publica.

[6] Bingham GM, Larrieu E, Uchiumi L, Mercapide C, Mujica G, Del Carpio M (2016). The economic impact of cystic Echinococcosis in Rio Negro Province, Argentina. Am J Trop Med Hyg.

[7] Larrieu E, Costa MT, Cantoni G, Labanchi JL, Bigatti R, Pérez A (2000). Control program of hydatid disease in the province of Río Negro Argentina. 1980–1997. Bol Chil Parasitol.

[8] Arezo M, Mujica G, Uchiumi L, Santillán G, Herrero E, Labanchi JL (2020). Identification of potential “hot spots” of cystic echinococcosis transmission in the province of Río Negro, Argentina. Acta Trop..

[9] Bingham GM, Budke CM, Larrieu E, Del Carpio M, Mujica G, Slater MR (2014). A community-based study to examine the epidemiology of human cystic echinococcosis in Rio Negro Province Argentina. Acta Trop.

[10] Salviti JC, Sobrino M, Del Carpio M, Mercapide C, Uchiumi L, Moguilensky J (2015). Hydatidosis: Ultrasonographyc screening in the Río Negro Province 25 years after the first screening. Acta Gastroenterol Latinoam.

[11] Larrieu E, Lester R, Rodriguez Jauregui J, Odriozzola M, Medina M, Agüero AM (1986). Epidemiology of human hydatidosis in the Province of Rio Negro, Argentina. Acta Gastroenterol Latinoam.

[12] Lawson A, Boaz R, Corberán-Vallet A, Arezo M, Larrieu E, Vigilato MA (2020). Integration of animal health and public health surveillance sources to exhaustively inform the risk of zoonosis: An application to echinococcosis in Rio Negro, Argentina. PLoS Negl Trop Dis..

[13] Del Carpio M, Mercapide CH, Salvitti JC, Uchiumi L, Sustercic J, Panomarenko H (2012). Early diagnosis, treatment and follow-up of cystic echinococcosis in remote rural areas in Patagonia: impact of ultrasound training of non-specialists. PLoS Negl Trop Dis.

[14] Brunetti E, Kern P, Vuitton DA (2010). Writing Panel for the WHO-IWGE. Expert consensus for the diagnosis and treatment of cystic and alveolar echinococcosis in humans. Acta Trop.

[15] Solomon N, Kachani M, Zeyhle E, Macpherson CNL (2017). The natural history of cystic echinococcosis in untreated and albendazole-treated patients. Acta Trop.

[16] Ziaei Hezarjaribi H, Fakhar M, Rahimi Esboei B, Soosaraei M, Ghorbani A, Nabyan N (2017). Serological evidence of human cystic echinococcosis and associated risk factors among general population in Mazandaran Province, northern Iran. Ann Med Surg.

[17] Larrieu E, Uchiumi L, Salvitti JC, Sobrino M, Panomarenko O, Tissot H (2019). Epidemiology, diagnosis, treatment and follow-up of cystic echinococcosis in asymptomatic carriers. Trans R Soc Trop Med Hyg.

[18] Andrabi A, Tak H, Lone BA, Para BA (2020). Seroprevalence of human cystic echinococcosis in South Kashmir, India. Parasite Epidemiol Control..

[19] Xue J, Zartarian V, Moya J, Freeman N, Beamer P, Black K (2007). A meta-analysis of children’s hand-to-mouth frequency data for estimating nondietary ingestion exposure. Risk Anal.

[20] Antwi-Agyei P, Biran A, Peasey A, Bruce J, Ensink J (2016). A faecal exposure assessment of farm workers in Accra, Ghana: a cross sectional study. BMC Public Health.

[21] Gorman Ng M, Davis A, van Tongeren M, Cowie H, Semple S (2016). Inadvertent ingestion exposure: hand- and object-to-mouth behavior among workers. J Expo Sci Environ Epidemiol.

[22] Cohen H, Paolillo E, Bonifacino R, Botta B, Parada L, Cabrera P (1998). Human cystic echinococcosis in a Uruguayan community: a sonographic, serologic, and epidemiologic study. Am J Trop Med Hyg.

[23] Moro PL, Cavero CA, Tambini M, Briceño Y, Jiménez R, Cabrera L (2008). Identification of risk factors for cystic echinococcosis in a peri-urban population of Peru. Trans R Soc Trop Med Hyg.

[24] Larrieu EJ, Costa MT, del Carpio M, Moguillansky S, Bianchi G, Yadon ZE (2002). A case-control study of the risk factors for cystic echinococcosis among the children of Rio Negro province, Argentina. Ann Trop Med Parasitol.

[25] Possenti A, Manzano-Román R, Sánchez-Ovejero C, Boufana B, La Torre G, Siles-Lucas M (2016). Potential risk factors associated with human cystic echinococcosis: systematic review and meta-analysis. PLoS Negl Trop Dis..

[26] Torgerson PR, Robertson LJ, Enemark HL, Foehr J, van der Giessen JWB, Kapel CMO (2020). Source attribution of human echinococcosis: a systematic review and meta-analysis. PLoS Negl Trop Dis.

[27] Casulli A, Tamarozzi F (2021). Tracing the source of infection of cystic and alveolar echinococcosis, neglected parasitic infections with long latency: the shaky road of “evidence” gathering. PLoS Negl Trop Dis.

[28] Tamarozzi F, Deplazes P, Casulli A (2020). Reinventing the wheel of *Echinococcus granulosus* sensu lato transmission to humans. Trends Parasitol.

