# Supplementary material for: Prevalence of human cystic echinococcosis in the towns of Ñorquinco and Ramos Mexia in Rio Negro Province, Argentina, and direct risk factors for infection
Source: Parasit Vectors. 2021 May 19;14:262. doi: 10.1186/s13071-021-04753-y (PMC8136178; doi:10.1186/s13071-021-04753-y)
Supplement: Supplementary file 1 — Additional file 1: Annex S1. Questionnaire. [file 13071_2021_4753_MOESM1_ESM.docx]

**Additional File S1 –QUESTIONNAIRE**

NAME OF THE STAFF ADMINISTERING THE QUESTIONNAIRE **___________________________________**

------------------------------------------------------------------------------------------------------------------------------------------

Village______________________________________________________Date ____________________

Name _____________________Surname_________________Sex ______Date of birth ______________

**PARTICIPANT CODE**

**RISK FACTORS QUESTIONNAIRES**

1) Do you have or you have had dogs in the past 5 years? YES NO

2) Do you touch dogs (independent if owned or not owned)? *Tick only one of the following options:*

NO, I never touch dogs

YES, **every day** **or frequently**

YES, **occasionally or rarely**

3) Do you eat vegetables that you/your family/friends grow (that is, not purchased in a shop/market)?

NO

YES, **every day or frequently**

YES, **occasionally or rarely**

4) Do you eat unwashed raw vegetables? *Tick only one of the following options:*

NO, I **only eat cooked** vegetables

NO, I **always wash** the vegetables when I will eat them uncooked

YES,I **always/almost always do not wash** vegetables when I eat them uncooked

YES, it **happens occasionally that I do not wash** vegetables when I eat them uncooked

4) Do you bite your nails? YES NO

5) Do you smoke? YES NO

6) Do you have the habit of keeping in the mouth a toothpick or a blade of grass? YES NO

7) Do you wash hands before preparing food?

NO, I **never wash hands** before preparing food

YES, I wash hands **every time or almost always** I prepare any food

YES, but only **occasionally** (before meals or when I remember/when I have water and soap at hands)

8) Do you wash hands before eating? *Tick only one of the following options:*

NO, I **never** was hands before eating

YES, I wash hands **every time or almost always** I eat, even what I am out and about

YES, but **only occasionally** (before meals or when I remember/when I have water and soap at hands)

9) What type of drinking water do you use? *Please provide the degree of use for every source option*

- Bottled/Commercial Always/Main source Occasionally Never
- House tap Always/Main source Occasionally Never
- Public place tap Always/Main source Occasionally Never
- Natural source Always/Main source Occasionally Never

(Example stream, lake, spring)

10) Where did you live your first 5 years of your life?

11) Do you have a relative with with CE disease?  NO YES: Who?______________
